# This Article Corrects: “Ileocecal Intussusception in the Adult Population: Case Series of Two Patients”

**DOI:** 10.5811/westjem.2022.11.59367

**Published:** 2022-11-07

**Authors:** Deena Ibrahim, Nina P. Patel, Malkeet Gupta, J Christian Fox, Shahram Lotfipour

**Affiliations:** * University of California, Irvine, Department of Emergency Medicine, Irvine, California; † Stanford University School of Medicine, Stanford California; ‡ University of California, Los Angeles, Department of Emergency Medicine, Westwood, California



*West J Emerg Med. 2010;197–200*.

Ileocecal Intussusception in the Adult Population: Case Series of Two Patients

Deena Ibrahim, Nina P. Patel, Malkeet Gupta, J Christian Fox, Shahram Lotfipour

[West J Emerg Med. 2022;23(6)958–958.] This article corrects a copyright issue with figure 1 in the original article.

